# Nontargeted Metabolomic Profiling of Huo-Tan-Chu-Shi Decoction in the Treatment of Coronary Heart Disease with Phlegm-damp Syndrome

**DOI:** 10.1155/2022/6532003

**Published:** 2022-08-12

**Authors:** Zhaoying Liang, Qiaohuang Zeng, Xiaomin Ou, Jing Cai, Taohua Lan, Weihui Lu

**Affiliations:** ^1^The Second Clinical Medical College of Guangzhou University of Chinese Medicine, Guangzhou 510405, China; ^2^State Key Laboratory of Dampness Syndrome of Chinese Medicine, The Second Affiliated Hospital of Guangzhou University of Chinese Medicine, Guangzhou 510020, China; ^3^Guangdong Provincial Hospital of Chinese Medicine, Guangzhou 510020, China; ^4^Guangdong Provincial Key Laboratory of Chinese Medicine for Prevention and Treatment of Refractory Chronic Diseases, Guangzhou 510020, China

## Abstract

**Background:**

Considered an effective supplementary therapy, traditional Chinese medicine (TCM) has been widely applied in the treatment of coronary heart disease (CHD). In this study, we aim to investigate the effects and mechanisms of Huo-Tan-Chu-Shi decoction (HTCSD, an in-hospital TCM prescription) in the treatment of CHD with the phlegm-damp syndrome in mice by non-targeted metabolomics with liquid chromatography-mass spectrometry (LC-MS)/MS.

**Methods:**

A CHD with phlegm-damp syndrome model was established with ApoE^−/−^ mice by subcutaneous injection with isoproterenol combined with high temperature, high humidity, and a high-fat diet, and divided into the HTCSD and Tanshi groups. C57BL/6 mice were set as the control group with an ordinary environment and diet. After administration, electrocardiogram (ECG), interventricular septum thickness (IVS) and left ventricular posterior wall thickness (LVPW), serum levels of creatine phosphokinase-Mb (CK-MB), cardiac troponin T (cTnT), lactic dehydrogenase (LDH) and oxidized low-density lipoprotein (oxLDL), and myocardial histopathological changes were recorded to assess myocardial damage. LC-MS/MS was applied to demonstrate the serum metabolic profile and explore potential mechanisms.

**Results:**

The obvious depressions of the ST segment and T wave presented in the ECG of Tanshi mice, while the depressions in ECG of HTCSD mice were significantly reduced. Compared with the control group, IVS, LVPW, and serum levels of CK-MB, cTnT, LDH, and oxLDL increased greatly in the Tanshi group, while these indicators decreased remarkably in the HTCSD group compared with those of the Tanshi group. Histopathology showed severe structural disorder, necrosis, and fibrosis of myocardial cells in Tanshi mice, which were alleviated in HTCSD mice. Metabonomics analysis showed obvious metabolic alterations among the experimental mice and revealed that the relevant metabolic pathways mainly included phospholipid metabolism, necroptosis, and autophagy.

**Conclusions:**

HTCSD has a certain therapeutic effect in mice with CHD with phlegm-damp syndrome via reducing myocardial ischemia, hypertrophy, and fibrosis. The underlying mechanisms involve the regulation of phospholipid metabolism, necroptosis, and autophagy.

## 1. Introduction

Coronary heart disease (CHD) also known as ischemic heart disease, is one of the leading causes of death worldwide, which seriously threatens human health. Traditional Chinese medicine (TCM), characterized as high safety and having multitargeted effects, has been considered a reliable alternative therapy and is extensively used in CHD treatment. In South China with a hot and humid climate, phlegm-damp syndrome, known as “Tanshi” in Chinese, is one of the major pathogenic factors of CHD [1]. Hence, Prof. Keji Chen (a famous TCM doctor) invented the Huo-Tan-Chu-Shi Decoction (HTCSD) based on Gualou-Xiebai series decoctions (the classical TCM formula used to treat CHD for thousands of years). HTCSD is made up of six Chinese herbal medicines, including Fructus Trichosanthis, Bulbus Allii Macrostemi, Rhizoma Pinelliae Praeparatum, Rhizoma Coptidis, Rhizoma Curcumae, and Radix Codonopsis. As an effective in-hospital prescription in the Guangdong Provincial Hospital of Chinese Medicine, previous research has found that several chemicals and CHD-related targets were regarded as the pivotal components and targets of HTCSD in the treatment of CHD [2]. On account of syndrome differentiation and overall adjustment of TCM treatment, further studies need to explore the underlying mechanism of HTCSD in treating CHD with phlegm-damp syndrome.

Metabolomics is a mature systematic biological technology in detecting and analyzing the overall and dynamic changes of endogenous metabolites, reflecting the biochemical process, and the physiological and pathological stages of the body [3]. It is analogous to the characteristics of multicomponent, multitarget, and holistic regulation of TCD treatment, which benefit the revelation of pharmacological mechanisms and pathways of TCM prescriptions [4]. Based on nontargeted metabolomics with liquid chromatography-mass spectrometry (LC-MS)/MS, this study aims to investigate the effect of HTCSD on serum metabolic profiling and the involved mechanisms in treating mice with CHD with phlegm-damp syndrome.

## 2. Methods

### 2.1. Materials and reagents

HTCSD contains the following Chinese herbal medicines: Fructus Trichosanthis 30 g, Bulbus Allii Macrostemi 30 g, Rhizoma Pinelliae Praeparatum 15 g, Radix Codonopsis 20 g, Rhizoma Curcumae 15 g, and Rhizoma Coptidis 6 g, which were purchased from Kangmei Pharmaceutical Co., Ltd (Guangdong, China). These herbs were soaked in 8-fold of water for 30 min, and then decocted twice for 30 min for each time, evaporated to a concentration of 1.16 g/mL, and stored at 4°C.

Isoproterenol (ISO) was obtained from Sigma-Aldrich (St. Louis, USA). Creatine phosphokinase-Mb (CK-MB), cardiac troponin T (cTnT), lactic dehydrogenase (LDH), and oxidized low-density lipoprotein (oxLDL) ELISA kits were purchased from Cusabio (Wuhan, China). Hematoxylin staining and eosin staining solutions were purchased from Yingjin Biotechnology Co., Ltd (Guangdong, China). Water, ethanol, acetonitrile, and formic acid were purchased from CNW Technologies GmbH (Düsseldorf, Germany). L-2-chlorophenylalanine was purchased from Shanghai Hengchuang Biotechnology Co., Ltd. (Shanghai, China).

All the chemicals and solvents were analytical or high-performance liquid chromatography grade.

### 2.2. Animal Model, Grouping, and Administration

Specific pathogen-free (SPF) male C57BL/6 and ApoE^−/−^ mice were provided by Beijing HFK Bioscience CO., LTD, and the animal experiment was approved by the Animal Care and Use Committee of the Guangdong Provincial Hospital of Chinese medicine. After 1 week of accommodation to the environment, ApoE^−/−^ mice were fed a western diet (21% fat, 1.5% cholesterol). After 4 weeks on a high-fat diet, ApoE^−/−^ mice were housed in a room at a temperature of 35 ± 0.5°C and a humidity of 90 ± 5% for 7 hours a day (the rest of the day in a room temperature of 20–25°C and a humidity of 50–60%). The mice were randomized into the Tanshi group and HTCSD group after 12 weeks of western diet and 8 weeks of high-temperature and high-humidity environment. C57BL/6 mice were housed in the laboratory environment (a temperature of 20–25°C and a humidity of 50–60%) and fed with a standard laboratory diet for 12 weeks as the control group. HTCSD mice were administered HTCSD intragastrically (23.2 g/kg/day) for 4 weeks, while the same volume of saline was given in the Tanshi and control group. ISO-induced myocardial ischemia is a classical model to explore the cardioprotective effects of various pharmacological interventions [5]. Subcutaneous injections with ISO (10 mg/kg/day) were executed in Tanshi and HTCSD mice for 7 days to simulate myocardial ischemia of CHD before euthanasia, while the control mice were given the same volume of saline.

### 2.3. Electrocardiography & Echocardiography

At 2 hours after the last ISO injection, electrocardiography was performed to record the limb lead II electrocardiogram (ECG) of the mice and analyze respectively the changes of the ST segment and T wave after 0 s, 1 min, 5 min, 10 min, and 20 min. Meanwhile, the echocardiogram equipped with the VisualSonics Vevo 2100 system and 21-MHz linear array transducer was applied to measure the left ventricular posterior wall thickness (LVPW) and interventricular septal thickness (IVS) via the parasternal short-axis view at the level of the papillary muscle by the M-mode tracing method.

### 2.4. Serum Biochemical Analysis & Histomorphology

After fasting for 12 hours, the mice were anesthetized with 20% urethane, and the eyeballs were removed for blood collection. The blood samples were placed in 1.5 mL EP tubes, centrifuged at 3500 rpm, and placed at 4°C for 10 minutes to collect serum samples. Enzymatic biochemical kits were used to separately determine the serum levels of CK-MB, cTnT, LDH, and oxLDL.

Mice hearts were separated and myocardial tissues with 0.5 cm in thickness were cut along the transverse axis in the middle of the left ventricle, fixing with 4% paraformaldehyde solution. The fixed tissues were embedded in paraffin wax and made into sections of 3.5 *μ*m thickness and subjected to hematoxylin & eosin (H&E) staining to observe the pathological changes.

All the protocols were followed in accordance with the manufacturer's recommendations.

### 2.5. Nontargeted LC-MS-Based Analysis

Serum samples of each group (*n* = 6) stored at −80°C were thawed at room temperature. 100 *μ*L of the sample was added to a 1.5 mL Eppendorf tube with 10 *μ*L of 2-chlorol-phenylalanine (0.3 mg/mL) and Lyso PC17 : 0 (0.01 mg/ml), respectively, dissolved in methanol as the internal standard, and the tube was vortexed for 10 s. Subsequently, 300 *μ*L of methanol and acetonitrile (2/1, v/v) was added, and the mixtures were vortexed for 1 min, ultrasonicated in an ice-water bath for 10 min, and stored at −20°C for 30 min. The extract was centrifuged at 13000 rpm, and placed at 4°C for 10 min. 300 *μ*L of the supernatant in a brown and glass vial was dried in a freeze concentration centrifugal dryer. 400 *μ*L mixture of methanol and water (1/4, v/v) were added to each sample, the samples were vortexed for 30 s, ultrasonicated for 3 min, and then placed at 4°C for 2 hours. Samples were centrifuged at 13000 rpm and placed at 4°C for 10 min. The supernatants (150 *μ*L) from each tube were collected using crystal syringes, filtered through 0.22 *μ*m microfilters, transferred to LC vials, and stored at −80°C.

A Dionex UltiMate 3000 RS UHPLC system fitted with a Q-Exactive Quadrupole-Orbitrap mass spectrometer and equipped with a heated electrospray ionization (ESI) source (Thermo Fisher Scientific, Waltham, MA, USA) was used to analyze the metabolic profiling in both ESI-positive and ESI-negative ion modes. An ACQUITY UPLC HSS T3 column (1.8 *μ*m, 2.1 × 100 mm) was employed in both the positive and negative modes. The binary gradient elution system consisted of (a) water (containing 0.1% formic acid, v/v) and (b) acetonitrile (containing 0.1% formic acid, v/v), and the separation was achieved using the gradient as shown in Supplementary Table S1. The flow rate was 0.35 mL/min and the column temperature was 50°C. All the samples were kept at 4°C during the analysis. The injection volume was 2 *μ*L. The mass range was from m/z 66.7 to 1,000.5. The resolution was set at 70,000 for the full MS scans and 35,000 for HCD MS/MS scans. The collision energy was set at 10, 20, and 40 eV. The mass spectrometer was operated as shown in Supplementary Table S2.

### 2.6. Data Processing and Statistical Analysis

The acquired LC-MS raw data were preanalyzed by the Progenesis QI software with ver2.3 (Nonlinear Dynamics, Newcastle, UK). Principle component analysis (PCA) and partial least squares-discriminant analysis (PLS-DA) were carried out to assess the general metabolic alterations among the three groups. A specific plot was used to present the number of differential metabolites among the groups. A heatmap and cluster plots were made to apparently exhibit the expression change of differential metabolites among the experimental groups. Metabolic pathways enrichment analysis was performed on differential metabolites based on the KEGG database. The selection of differential metabolites was based on a variable influence on the projection (VIP) values obtained from the OPLS- DA model and the *p* values from a two-tailed Student's *t*-test. Metabolites with VIP values larger than 1.0 and *p* values less than 0.05 were regarded as differential metabolites.

In vivo data were presented as mean ± standard deviation and statistical analysis between groups was performed by one-way analysis of variance with the LSD test via the GraphPad Prism software v7.0. *P* values less than 0.05 were considered statistically significant.

## 3. Results

### 3.1. HTCSD Reduced Myocardial Injury in Mice Induced by the Administration of ISO Combined with High Temperature and High Humidity, and a High-Fat Diet

**Figure 1:**
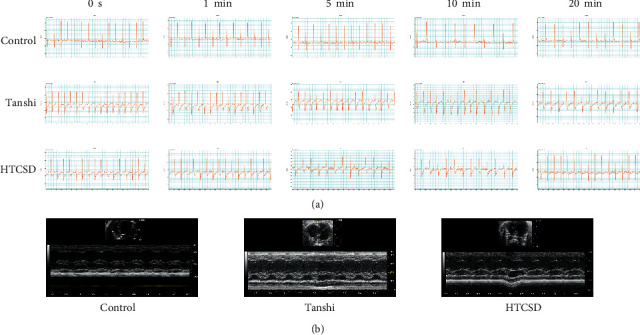
Electrocardiography and echocardiography. (a) ECGs of limb lead II of mice were recorded to evaluate the changes of the ST segment and T wave. (b) LVPW and IVS of mice were measured by echocardiography. Abbreviations: ECG: electrocardiogram, LVPW: left ventricular posterior wall thickness, IVS: interventricular septal thickness, HTCSD : Huo-Tan-Chu-Shi decoction.

From the results of ECG and echocardiography, as shown in Figure 1, 1(a) and Table 1&2, the significant depressions of the ST segment and T wave were presented in the ECG of the Tanshi group compared with that of the control group, while the depressions in ECG of the HTCSD group mitigated remarkably. As presented in Figure 1, 1(b) and Table 3, compared with the control group, IVS and LVPW at systole and diastole increased greatly in the Tanshi group, while these values obviously improved in the HTCSD group. These data indicated that critical myocardial ischemia and hypertrophy arose in Tanshi mice, and HTCSD can effectively ameliorate the ischemic damage and pathological hypertrophy of the myocardium.

**Figure 2:**
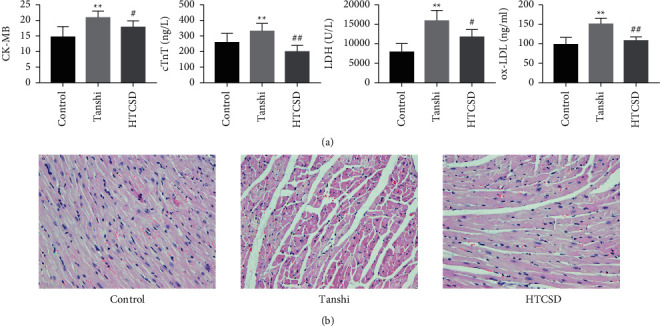
Serum biochemical analysis and histomorphology. (a) Serums were collected to evaluate the levels of CK-MB, cTnT, LDH, and oxLDL and are shown as mean ± *S*.D (*n* = 5–6). ^*∗∗*^*P*-value<0.01 (Tanshi vs Control), ^##^*P*-value<0.01 (HTCSD vs Tanshi), and ^#^*P*-value<0.05 (HTCSD vs Tanshi). (b) H&E staining (scale bar = 50 *μ*m) was used to observe the pathological changes of the myocardium of the left ventricle. Abbreviations: CK-MB: creatine phosphokinase-Mb, cTnT: cardiac troponin T LDH: lactic dehydrogenase, oxLDL: oxidized low-density lipoprotein, H&E: hematoxylin and eosin. HTCSD: Huo-Tan-Chu-Shi decoction.

Besides, as shown in Figure 2, 2(a), the serum levels of CK-MB, cTnT, LDH, and oxLDL elevated significantly in Tanshi mice compared with those of control mice, while decreases in various degrees were found in HTCSD mice compared with Tanshi mice. Furthermore, as presented in (Figure 2, 2(b)) by H&E staining, the myocardial cells in the control mice had normal morphology and a structure with a distinct texture. However, notably, the structure disordered of myocardial cells with inflammatory cells infiltration, extensive swelling, and rupture of myocardial fibers, and dramatic edema of the intercellular spaces were found in Tanshi mice. In HTCSD mice, the myocardial pathological changes improved with the mitigation of myocardial fiber rupture, inflammatory infiltration, and tissue edema. These results suggested that Tanshi mice suffered from severe myocardial injury and fibrosis, while the damage was improved with the treatment of HTCSD.

Taken together, our study demonstrated that the application of ISO combined with high temperature and high humidity, and a high-fat diet induced severe myocardial injury in mice. In addition, HTCSD showed a certain therapeutic effect in mice with CHD with phlegm-damp syndrome by reducing myocardial ischemia, hypertrophy, and fibrosis.

### 3.2. Analysis of Serum Metabolomic Profiling

**Figure 3:**
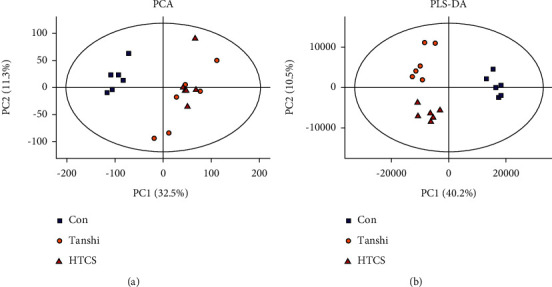
Score plots of PCA and PLS-DA. (a) Score plot of principle component analysis (PCA). (b) Score plot of partial least squares-discriminant analysis (PLS-DA). Abbreviations: HTCSD : Huo-Tan-Chu-Shi decoction.

In this study, score plots of PCA and PLS-DA were used to generally evaluate the metabolic alterations. As presented in Figures 3, 3(a)& 3(b), the score plots of PCA manifested a complete distinction between the control and Tanshi groups, and an overlap was observed between the Tanshi and HTCSD groups. PLS-DA showed apparent separations among the control, Tanshi, and HTCSD groups. The results showed a significant difference of the metabolic profiling among the three groups.

**Figure 4:**
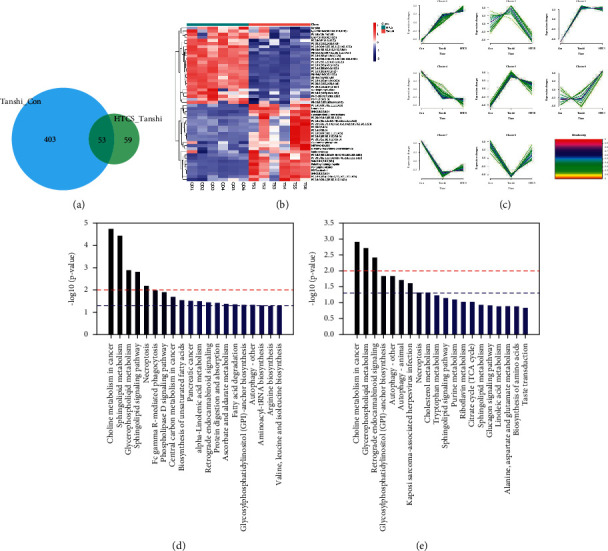
Serum metabolism profile alterations and metabolite-associated pathways. (a) The amount of differential metabolites among the experimental groups. (b) Heatmap of top 50 differential metabolites in the HTCSD vs Tanshi group. (c) Clusters of time sequence analysis of differential metabolites among the experimental groups. (d) The column chart of the top 20 KEGG pathways in the Tanshi vs control group. (e) The column chart of the top 20 KEGG pathways in the HTCSD vs Tanshi group. Abbreviations: HTCSD : Huo-Tan-Chu-Shi decoction.

Considering the screening criterion of VIP >1 and *P* < 0.05, 456 differential metabolites were found between the control and Tanshi groups, 112 differential metabolites between the Tanshi and HTCSD groups, and 53 differential metabolites in common as shown in Figure 4, 4(a). According to VIP, the top 50 differential metabolites between the HTCSD and Tanshi groups were shown in heatmaps (Figure 4, 4(b)). The color from blue to red indicates the expression of metabolites from low to high, and there was a remarked differentiation between HTCSD and Tanshi groups. Furthermore, on the basis of the theory of the R package Mfuzz fuzzy clustering, differential metabolites in common among the three groups were collected into several clusters, and then, time sequence analysis was performed for observing the variation trend. Figures 4, 4(c), and Table 4 show differential metabolites of clusters 2, 5, and 8. In Table 4, the value of membership indicatesthe degree of differential metabolites conform with the relevant cluster. The metabolites 

with membership> 0.4 were select for further analysis, the superclass of which was mainly lipids and lipid-like molecules.

To comprehensively investigate metabolic disturbances among experimental groups, metabolic pathway analyses were performed by the KEGG database. The column charts of the top 20 KEGG pathways in the Tanshi vs control group and the HTCSD vs Tanshi group are presented in Figures 4, 4(d) and 4, 4(e). Several same pathways were found among three groups, mainly including glycerophospholipid metabolism, sphingolipid metabolism, necroptosis, and autophagy. Combing the selected differential metabolites in Table 4 with the metabolic pathways in common, the molecule mechanism of HTCSD in the treatment of CHD with phlegm-damp syndrome may involve the regulation of lipid metabolism, necroptosis, and autophagy.

## 4. Discussion

TCM combined with modern medicine has become an effective therapeutic strategy and is widely applied in China [6]. In addition to the therapeutic concept of holism and syndrome differentiation, TCM emphasizes the treatment in accordance with local conditions. With the hot and humid climate in South China, spleen qi deficiency and phlegm-damp syndrome are considered the main types of TCM constitutions in the local people [7]. With the effect of improving qi, promoting blood circulation, resolving phlegm, and eliminating dampness, HTCSD was used to treat patients with CHD with phlegm-damp syndrome.

In our study, the administration of ISO was applied to stimulate myocardial ischemia. The changes of the ST segment and T wave of ECG, biochemical indexes such as cTnT, CK-MB, and LDH, and histopathology are crucial evaluation indicators for myocardial ischemia and cell injury. Meanwhile, high temperature, high humidity, and a high-fat diet were adopted to induce phlegm-damp syndrome. As shown in the experimental results, we built a CHD with phlegm-damp syndrome model in mice. And, we demonstrated that the Chinese herbal formula HTCSD has a therapeutic effect in reducing myocardial ischemia, hypertrophy, and fibrosis in mice. Based on metabolomic analysis, the associated mechanisms primarily included phospholipid metabolism, cell necroptosis, and autophagy.

### 4.1. Regulation of Phospholipid Metabolism

As shown in this study, phospholipids are the main type of lipids expressing abnormally among the experimental mice. Phospholipids mainly include glycerophospholipids and sphingolipids. Among them, glycerophospholipids with high content in the body include phosphatidylethanolamine (PE), phosphatidylcholine (PC), phosphatidylserine (PS), and phosphatidylinositide (PI) [8], of which PC and PE were considered as the potential biomarkers of cardiovascular diseases [9, 10]. With the effect of phospholipase A2, PC transforms into lysophosphatidylcholine (LysoPC), which is the main component of oxLDL [11]. In our study, it was found that the serum levels of PC (14 : 0) and PC (O-16 : 0) increased while PC (16 : 1), PC (18 : 0), PC (18 : 1), PC (20 : 2), LysoPC (18 : (2), and LysoPC (20 : (4) decreased in Tanshi mice compared with the control group. And, the treatment of HTCSD downregulated the PC (14 : 0) and PC (O-16 : 0) levels while upregulating the serum levels of PC (16 : 1), PC (18 : 0), PC (18 : 1), PC (20 : 2), LysoPC (18 : (2), and LysoPC (20 : 4), which is in accordance with the result of a previous study [12]. Based on the type of cell and the stage of inflammatory response and oxidizing reaction, LysoPC plays various roles in the progression of AS [13–15]. Research has shown that LysoPC induces the migration of lymphocyte and macrophage and the activation of oxidative stress, and increases proinflammatory cytokines levels, accelerating the development of CHD [16, 17]. However, some types of LysoPC show a negative association with CHD [10], which can inhibit the synthesis and foaming of macrophages and reduce cholesterol accumulation [18]. In addition, PE is associated with oxidative phosphorylation, the stability of mitochondrial, and autophagy [19]. A study found that PE containing unsaturated fatty acids increases the risk of CHD [20]. Our present study showed that the concentration of PE(0 : 0/22 : 6(4Z,7Z,10Z,13Z,16Z,19Z)) and PE(O-18 : 1(9Z)/0 : 0) elevated in mice with CHD with phlegm-damp syndrome the administration of HTCSD was reduced. Furthermore, PS was proved to have an atheroprotective effect by regulating cholesterol metabolism, inhibiting inflammatory response, and enhancing the function of high-density lipoprotein (HDL) [21]. This study presented that PS(P-18 : 0/0 : 0) and PS(20 : 4(5Z,8Z,11Z,14Z)/19 : 0) levels decreased in Tanshi mice, while increased in HTCSD mice. As mentioned previously, with the regulation of glycerophospholipids, HTCSD may improve coronary AS in mice by modulating the function of immune cells and cholesterol metabolism, inhibiting oxidative stress and inflammatory response. Sphingolipid metabolism is another phospholipid-associated pathway found in our study. As the key of sphingolipid metabolism, ceramide can form sphingomyelin (SM) with the incorporation of phosphocholine. And, ceramide can be metabolized to generate sphingosine (sph), which can further phosphorylate to form sph 1-phosphate (S1P) [22]. Studies have shown that both SM and S1P can induce inflammatory responses of smooth muscle cells in the coronary artery, and SM is associated with atherosclerotic plaque instability [23]. In addition, it has been reported that the S1P bond to HDL has antiatherosclerotic effects, while the S1P bond to non-HDL is negatively associated with CHD [24]. Our present study shows that the levels of SM (d18 : 0/16 : 0) and sph elevated in Tanshi mice, while decreased after medication. This study suggested that HTCSD might reduce smooth muscle cell inflammation and enhance plaque stability via downregulating the levels of SM and sph.

Collectively, with the regulation of glycerophospholipids and sphingolipids, HTCSD can improve the disorder of lipid profile and finally mitigate myocardial injury in mice. Previous research proved that lipids are associated with the occurrence and progression of CHD [25, 26]. In addition, a study indicated that patients with CHD with phlegm syndrome were prone to have inordinate lipid metabolism, and the medication of Gualou-Xiebai-Banxia decoction (one of the Gualou-Xiebai series decoctions) promoted the improvement of lipid metabolism and the stability of the cell membrane [27]. As the herbs reserved from the classical prescription, Fructus Trichosanthis, Bulbus Allii Macrostemi, and Rhizoma Pinelliae Praeparatum in HTCSD have a dramatic antihyperlipidemia effect by regulating the lipid level and inhibiting lipid accumulation [28]. With the improvement of lipid metabolism, these herbs protect the damaged myocardium.

### 4.2. Regulation of Cell Necroptosis

In this study, we found that necroptosis is another important metabolic pathway involved in the treatment of CHD with phlegm-damp syndrome by HTCSD. With elaborate regulation by the intracellular signaling molecular pathways, necroptosis is induced by a series of death receptors (including tumor necrosis factor receptors (TNFR) 1, 2 and fatty acid synthase) and activated by the formation of a receptor-interacting protein (RIPK) 1/RIPK3 necrosome. The further combination with the RIPK 1/RIPK3 necrosome and mixed lineage kinase domain-like (MLKL) protein forms a necrotic complex, which mediates the process of necroptosis [29]. Necroptosis conducts cytolysis and contributes to severe inflammatory responses on account of the rapid loss of the plasma membrane integrity and the release of intracellular proinflammatory contents, which is considered an important pathological and physiological procedure of ischemia-reperfusion injury, myocardial infarction, and cardiac remodeling [30]. In early AS, vascular monocytes phagocytose modified lipoproteins (such as oxLDL) to form macrophage foam cells, which further increases the expression of RIP3 and MLKL in macrophage foam cells, inducing inflammatory responses and aggravating AS progression [31]. The expression of RIP3 in myocardial cells mediates the production of inflammation and reactive oxygen species and the pathological myocardial remodeling [32]. In addition, the inhibition of RIP1 can reduce the area of myocardial infarction after induction of ischemia [33]. As shown in our study, excessive necroptosis might generate in mice of the Tanshi group, inducing wide inflammation and myocardial damage. With the regulation of necroptosis, HTCSD may inhibit inflammatory responses and AS progression in mice by downregulating the serum level of oxLDL and the expressions of RIP3 and MLKL.

### 4.3. Regulation of Autophagy

Autophagy is also one of the underlying mechanisms involved in the pathological and medication process. The molecular pathway of autophagy mainly includes a mammalian target of rapamycin (mTOR), adenosine monophosphate-activated protein kinase (AMPK), endoplasmic reticulum stress, and p53 [34]. Activated as a cytoprotective procedure, autophagy promotes the release of nutrients from amino acids, fatty acids, and monosaccharides into the cytoplasm, resulting in the recycling of cytoplasmic components for protein synthesis and adenosine triphosphate (ATP) production [35, 36]. Research has demonstrated that autophagy has an atheroprotective effect via inhibiting inflammation and apoptosis, promoting cholesterol efflux, and reducing lipid deposition, while the dysfunction of autophagy exacerbates AS progression [37]. With the lack of sufficient glucose, amino acids, and energy, the ischemia-induced autophagy response is initiated by activating AMPK and inhibiting mTOR signaling, showing a cardioprotection effect [38–40]. In contrast, under the circumstance of a high-fat diet, the maturation of autophagy is disrupted in myocardial cells and the cardioprotection effect is diminished [41]. Moreover, a study has proven that damp-heat syndrome can lead to the dysfunction of autophagy in atherosclerotic rats. Berberine, the main active ingredient in Rhizoma Coptidis (one of the herbs of HTCSD), was found to have an atheroprotective effect by enhancing plaque stability, the mechanism which may be involved with the regulation of autophagy [42, 43]. In our study, it is found that the autophagy activity may decrease in mice with CHD with phlegm-damp syndrome. The administration of HTCSD may improve the autophagy activity via the AMPK/mTOR signaling pathway, resulting in inhibiting inflammatory reactions and enhancing plaque stability.

## 5. Limitation

Our study has some limitations. First, metabolomics data are massive and complex. The number and variety of metabolites identified in serum may differ from cells or tissues. Second, a small sample was adopted in our study and there are only few metabolomics studies of TCM treatment in CHD. Some experiment results were inconsistent and difficult to determine. In summary, this study was a preliminary exploration of the mechanism of HTCSD via nontargeted metabolomics, extensive research is needed to investigate the deeper molecular mechanisms.

## 6. Conclusion

In conclusion, this study demonstrated that HTCSD alleviated myocardial ischemia, hypertrophy, and fibrosis in mice. The mechanism involved may be as followed: regulation of phospholipid metabolism, necroptosis, and autophagy. It provides directions for further investigations to excavate the more precise molecular mechanisms. [37–42]

## Figures and Tables

**Table 1:** Changes of the ST segment in the ECG of mice (mean ± *S*.D, *n* = 5–6).

ST height (mV)					
	0s	1 min	5 min	10 min	20 min
Control	0.1118 ± 0.0383	0.0991 ± 0.0394	0.1145 ± 0.0357	0.1531 ± 0.0399	0.1456 ± 0.0453
Tanshi	−0.2387 ± 0.0450^*∗∗*^	−0.2557 ± 0.0614^*∗∗*^	−0.2321 ± 0.0958^*∗∗*^	−0.2291 ± 0.0977^*∗∗*^	−0.2062 ± 0.0694^*∗∗*^
HTCSD	−0.1608 ± 0.0393^#^	−0.1148 ± 0.0781^##^	−0.1305 ± 0.0551^#^	−0.1169 ± 0.0438^#^	−0.0800 ± 0.0413^##^

Note:^*∗∗*^*P*-value<0.01 (Tanshi vs Control), ^#^*P*-value<0.05 (HTCSD vs Tanshi), and ^##^*P*-value<0.01 (HTCSD vs Tanshi). ECG: electrocardiogram, HTCSD: Huo-Tan-Chu-Shi decoction.

**Table 2:** Changes of *T* wave in the ECG of mice (mean ± *S*.D, *n* = 5–6).

T Amplitude (mV)					
	0s	1 min	5 min	10 min	20 min
Control	0.1289 ± 0.0291	0.1133 ± 0.0313	0.1270 ± 0.0357	0.1415 ± 0.0355	0.1638 ± 0.0413
Tanshi	−0.1980 ± 0.0265^*∗∗*^	−0.2169 ± 0.0460^*∗∗*^	−0.2276 ± 0.0722^*∗∗*^	−0.2256 ± 0.0538^*∗∗*^	−0.1838 ± 0.0578^*∗∗*^
HTCSD	−0.1494 ± 0.0419	−0.1297 ± 0.0738^#^	−0.1250 ± 0.0489^##^	−0.1218 ± 0.0558^##^	−0.1161 ± 0.0748

Note:^*∗∗*^*P*-value<0.01 (Tanshi vs Control), ^#^*P*-value<0.05 (HTCSD vs Tanshi), and ^##^*P*-value<0.01 (HTCSD vs Tanshi). ECG: electrocardiogram, HTCSD: Huo-Tan-Chu-Shi decoction.

**Table 3:** Comparison of LVPW and IVS measured by echocardiography (mean ± *S*.D, *n* = 5–6).

	IVSD (mm)	IVSS (mm)	LVPWD (mm)	LVPWS (mm)
Control	0.88 ± 0.24	1.35 ± 0.34	1.14 ± 0.30	1.50 ± 0.29
Tanshi	1.20 ± 0.10^*∗∗*^	2.08 ± 0.35^*∗∗*^	1.68 ± 0.24^*∗∗*^	2.03 ± 0.44^*∗*^
HTCSD	0.91 ± 0.26^#^	1.83 ± 0.17	1.28 ± 0.27^#^	1.51 ± 0.33^#^

Note:^*∗*^*p*-value<0.05 (Tanshi vs Control), ^*∗∗*^*P*-value<0.01 (Tanshi vs Control), and ^#^*P*-value<0.05 (HTCSD vs Tanshi). IVSD: interventricular septal thickness at diastole, IVSS: interventricular septal thickness at systole, LVPWD: left ventricular posterior wall thickness at diastole, LVPWS: left ventricular posterior wall thickness at systole, ECG: electrocardiogram, HTCSD: Huo-Tan-Chu-Shi decoction.

**Table 4:** Differential metabolites of cluster 2, cluster 5, and cluster 8 (membership>0.4).

Cluster number	Metabolites	Membership
Cluster 2	FAD	0.710145334
Cluster 2	Fucoxanthinol 3-myristoleate	0.693372847
Cluster 2	PE(0 : 0/22 : 6(4Z,7Z,10Z,13Z,16Z,19Z))	0.664223572
Cluster 2	SM(d18 : 0/16 : 0)	0.450356799
Cluster 2	Cadusafos	0.416098027
Cluster 2	(3Z)-2-propylpent-3-enoic acid	0.401266799
Cluster 5	5-Deoxydiplosporin	0.859002946
Cluster 5	Inosine triphosphate	0.853761932
Cluster 5	SLF	0.839544464
Cluster 5	Garcinia lactone dibutyl ester	0.835935897
Cluster 5	9-Hydroperoxy-12,13-dihydroxy-10-octadecenoic acid	0.832606958
Cluster 5	PC(14 : 0/20 : 3(8Z,11Z,14Z))	0.806742973
Cluster 5	2,5-Octadien-1-ol	0.784832227
Cluster 5	3-Methyl-3-butenyl apiosyl-(1->6)-glucoside	0.68681373
Cluster 5	Histidine-Phenylalanine	0.665285993
Cluster 5	5,10-Methenyltetrahydrofolate	0.658926736
Cluster 5	3a,21-Dihydroxy-5b-pregnane-11,20-dione	0.633962294
Cluster 5	(2-Methyl-3-phenylpropoxy)sulfonic acid	0.632564399
Cluster 5	Zizyphine A	0.616615337
Cluster 5	DG(22 : 4(7Z,10Z,13Z,16Z)/24 : 0/0 : 0)	0.615378609
Cluster 5	Militarinone B	0.610631061
Cluster 5	PE(O-18 : 1(9Z)/0 : 0)	0.596672725
Cluster 5	Lipoxin D4	0.580309909
Cluster 5	Sphingosine	0.57731702
Cluster 5	PC(O-16 : 0/0 : 0)	0.564222357
Cluster 5	Uralenneoside	0.56046916
Cluster 5	Hoffmanniolide	0.552641588
Cluster 5	4-(2,6,6-Trimethyl-1-cyclohexenyl)-2-butanol	0.552081026
Cluster 5	Tetraneurin A	0.531319613
Cluster 5	2-O-(beta-D-galactopyranosyl-(1->6)-beta-D-galactopyranosyl) 2S,3R-dihydroxynonanoic acid	0.511911474
Cluster 5	Terbutaline-1-sulfate	0.500476178
Cluster 5	OKDdiA-PE	0.498764561
Cluster 5	Valorphin	0.480345113
Cluster 5	PHOHA-PS	0.466379377
Cluster 5	Galabiosylceramide (d18 : 1/24 : 1(15Z))	0.465226025
Cluster 5	((3,4,5-Trihydroxy-6-(1,2,6-trihydroxy-3-(hydroxy(3,4,5-trihydroxyoxan-2-yl) methyl)-4-oxocyclohexa-2,5-dien-1-yl)oxan-2-yl) methoxy)sulfonic acid	0.460888285
Cluster 5	15-Cyclohexyl pentanor PGF2alpha	0.45349852
Cluster 5	Tryptophyl-asparagine	0.434188447
Cluster 5	Beauvericin	0.418981588
Cluster 5	9,10-Dihydroxy-13-hydroperoxy-11-octadecenoic acid	0.408170734
Cluster 5	11-Deoxy-11-methylene-PGD2	0.402497413
Cluster 5	OKOOA-PC	0.402386826
Cluster 8	PC(18 : 0/P-18 : 1(11z))	0.762565962
Cluster 8	PS(P-18 : 0/0 : 0)	0.751504134
Cluster 8	N-Methyl-a-aminoisobutyric acid	0.718517348
Cluster 8	Rutagravine	0.701523565
Cluster 8	PS(20 : 4(5Z,8Z,11Z,14Z)/19 : 0)	0.690096004
Cluster 8	Streptidine	0.574309645
Cluster 8	LysoPC(20 : 4(8Z,11Z,14Z,17Z))	0.567252783
Cluster 8	PC(18 : 1(9Z)/P-18 : 1(9z))	0.508347134
Cluster 8	LysoPC(18 : 2(9Z,12Z))	0.477289213
Cluster 8	PC(16 : 1(9Z)/18 : 1(9z))	0.437316248
Cluster 8	PC(20 : 2(11Z,14Z)/14 : 0)	0.428489908
Cluster 8	31-Hydroxy rifabutin	0.423115022

Note: The value of membership indicates the degree of differential metabolites conform with the relevant cluster.

## Data Availability

All relevant data are accessible from the corresponding author (e-mail: weihui.lu@gzucm.edu.cn).

## Supplementary Materials

Supplementary Materials Supplementary Table S1. The elution gradient. Supplementary Table S2. The mass spectrometry parameter.
