# Supplementary material for: Nontargeted Metabolomic Profiling of Huo-Tan-Chu-Shi Decoction in the Treatment of Coronary Heart Disease with Phlegm-damp Syndrome
Source: Cardiol Res Pract. 2022 Aug 12;2022:6532003. doi: 10.1155/2022/6532003 (PMC9391147; doi:10.1155/2022/6532003)
Supplement: Supplementary Materials — Supplementary Table S1. The elution gradient. Supplementary Table S2. The mass spectrometry parameter. [file 6532003.f1.docx]

**Supplementary Table S1. The elution gradient.**

| **Time（min）** | **A（%）** | **B（%）** |
| --- | --- | --- |
| 0 | 95 | 5 |
| 1 | 95 | 5 |
| 12 | 0 | 100 |
| 16 | 0 | 100 |
| 16.1 | 95 | 5 |
| 18 | 95 | 5 |

**Supplementary Table S2. The mass spectrometry parameter.**

| **Parameter** | **positive ion mode** | **negative ion mode** |
| --- | --- | --- |
| Spray Voltage (V) | 3800 | -3500 |
| Capillary Temperature (°C) | 320 | 320 |
| Aux gas heater temperature (℃) | 350 | 350 |
| Sheath Gas Flow Rate (Arb) | 35 | 35 |
| Aux gas flow rate (Arb) | 8 | 8 |
| S-lens RF level | 50 | 50 |
| Mass range (m/z) | 100-1000 | 100-1000 |
| Full ms resolution | 70000 | 70000 |
| MS/MS resolution | 17500 | 17500 |
| NCE/stepped NCE | 10, 20, 40 | 10, 20, 40 |
